# Detection of Emerging Vaccine-Related Polioviruses by Deep Sequencing

**DOI:** 10.1128/JCM.00144-17

**Published:** 2017-06-23

**Authors:** Malaya K. Sahoo, Marisa Holubar, ChunHong Huang, Alisha Mohamed-Hadley, Yuanyuan Liu, Jesse J. Waggoner, Stephanie B. Troy, Lourdes Garcia-Garcia, Leticia Ferreyra-Reyes, Yvonne Maldonado, Benjamin A. Pinsky

**Affiliations:** a Department of Pathology, Stanford University School of Medicine, Stanford, California, USA; b Department of Medicine, Division of Infectious Diseases and Geographic Medicine, Stanford University School of Medicine, Stanford, California, USA; c Department of Pediatrics, Division of Infectious Diseases, Stanford University School of Medicine, Stanford, California, USA; d Eastern Virginia Medical School, Norfolk, Virginia, USA; e Instituto Nacional de Salud Pública, Cuernavaca, Morelos, Mexico; Boston Children's Hospital

**Keywords:** poliovirus, high-throughput nucleotide sequencing, poliovirus vaccine, oral, enterovirus, molecular methods, oral vaccines

## Abstract

Oral poliovirus vaccine can mutate to regain neurovirulence. To date, evaluation of these mutations has been performed primarily on culture-enriched isolates by using conventional Sanger sequencing. We therefore developed a culture-independent, deep-sequencing method targeting the 5′ untranslated region (UTR) and P1 genomic region to characterize vaccine-related poliovirus variants. Error analysis of the deep-sequencing method demonstrated reliable detection of poliovirus mutations at levels of <1%, depending on read depth. Sequencing of viral nucleic acids from the stool of vaccinated, asymptomatic children and their close contacts collected during a prospective cohort study in Veracruz, Mexico, revealed no vaccine-derived polioviruses. This was expected given that the longest duration between sequenced sample collection and the end of the most recent national immunization week was 66 days. However, we identified many low-level variants (<5%) distributed across the 5′ UTR and P1 genomic region in all three Sabin serotypes, as well as vaccine-related viruses with multiple canonical mutations associated with phenotypic reversion present at high levels (>90%). These results suggest that monitoring emerging vaccine-related poliovirus variants by deep sequencing may aid in the poliovirus endgame and efforts to ensure global polio eradication.

## INTRODUCTION

Since the Global Polio Eradication Initiative's inception in 1998, paralysis due to wild poliovirus has declined by >99% from an estimated 350,000 cases in 1988 to 74 reported cases in 2015 (1). This success is due in large part to the widespread use of Sabin oral poliovirus vaccine (OPV), which has been the backbone of polio eradication efforts worldwide due to its ease of administration, immunogenic potential, and low cost (2). However, OPV itself causes vaccine-associated paralytic poliomyelitis, which is estimated to occur at a rate of 2 to 4 cases/1,000,000 live births per year in countries using OPV (3). In addition, OPV can develop into vaccine-derived polioviruses (VDPVs), mutated vaccine strains with the potential to behave like wild-type neurovirulent poliovirus. Circulating VDPVs (cVDPVs), those with evidence of community transmission, are phenotypically indistinguishable from wild poliovirus and have caused outbreaks in at least 18 countries since 2000 (4). VDPVs, and in particular cVDPVs, jeopardize years of polio eradication efforts.

OPV strains accumulate mutations over time via replication in a vaccinated individual's gut and/or transmission within a community. However, vaccine-related viruses are categorized as VDPVs only if there is significant genetic divergence from its parent strain, representing prolonged (>1-year) excretion or community transmission. Sequencing of the region encoding the VP1 capsid protein is used to compare a vaccine-related virus to its parent; the amount of divergence required to meet the definition of VDPV differs by serotype (>1% divergence for serotypes 1 and 3 and >0.6% divergence for serotype 2) (5). However, the evolution of vaccine strains into VDPVs is not well characterized. Most data regarding vaccine-related polioviruses and VDPVs are from samples collected during investigations of outbreaks triggered by acute flaccid paralysis cases or from environmental surveillance (6–11). A few studies have sequenced vaccine-related viruses isolated from asymptomatic individuals or their contacts after OPV vaccination; however, these studies relied upon tissue culture amplification, which may on its own introduce mutations, and Sanger sequencing technology, which is insensitive to low-level variants (12–15).

In a previous prospective cohort study, we evaluated OPV shedding in the stool of vaccinated, asymptomatic children and their close contacts (16). In this study, we describe a culture-independent, deep-sequencing methodology and informatics pipeline to characterize the OPV strains shed by this cohort.

(A portion of this work was previously presented at the Association for Molecular Pathology Annual Meeting, 13 to 15 November 2014, Washington, DC.)

## RESULTS

### Determining the error threshold for the characterization of vaccine-related polioviruses.

To characterize the error present in our sequencing approach, reverse transcription (RT)-PCR targeting the poliovirus 5′ UTR and P1 region, encoding the structural viral proteins VP1 to VP4, was carried out using extracted RNA from low-passage-number control Sabin serotype 1, 2, and 3 strains (Sabin 1, 2, and 3, respectively) as the template. The variants detected from the sequencing data of these controls were considered noise and used to set an error threshold that limits variant false discovery.

**FIG 1:**
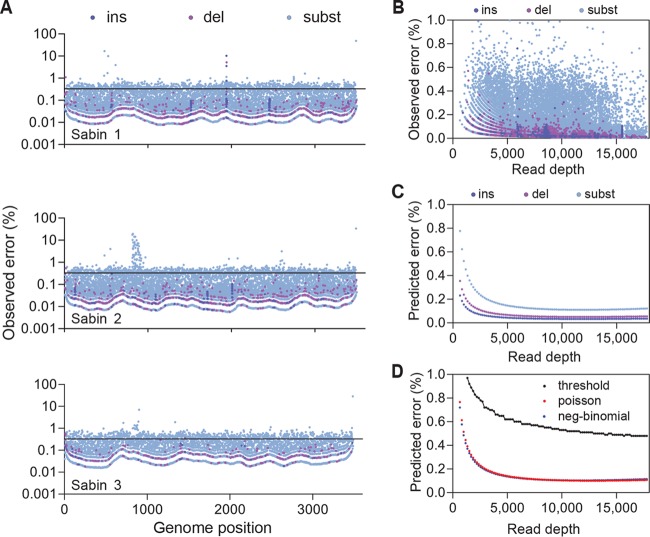
Determination of the error threshold. (A) Error events observed across the genome in low-passage-number controls. Insertions (ins), deletions (del), and substitutions (subst) are color coded. Note the short error-prone region (bp 810 to 900) in Sabin 2. The solid black line represents the overall per base error rate, 0.0033. (B) Error events plotted as a function of read depth. (C) Prediction of insertion, deletion, and substitution error rates based on sequence depth using a general linear model (Poisson) with observed error data. (D) Comparison of overall errors using Poisson and negative-binomial (gamma-Poisson mixture) models. The black curve represents the error threshold utilized in this study. The threshold was calculated from the overall per base error rate using the Poisson distribution at a *P* of <0.001.

While the Sabin 1, Sabin 2, and Sabin 3 controls had comparable distributions of error, we identified a short error-prone region in Sabin 2 (positions 810 to 900) that was not present in either Sabin 1 or Sabin 3 (Fig. 1A). This error-prone region was confirmed by deep sequencing a plasmid clone of the Sabin 2 amplicon and was not present when a plasmid clone of the Sabin 1 amplicon was sequenced (data not shown). Overall, the observed per base error rate in the low-passage-number controls (0.0033) was similar to the observed per base error rate in the cloned plasmids (0.0037), suggesting that the variants identified in these experiments arose primarily from the technical aspects of sample preparation and sequencing rather than from low-passage-number culture.

To further characterize the error in our sequencing protocol, we utilized the observed error (Fig. 1B) to model the central tendencies of the method noise using a general linear or Poisson model (Fig. 1C). This analysis demonstrated that the errors were more likely to be substitutions rather than insertions or deletions (Fig. 1C). Homopolymers had no effect on error rates (data not shown). We also compared the error rates predicted by the Poisson model with the error rates predicted by the negative-binomial model, also referred to as a gamma-Poisson mixture (Fig. 1D). Both models predicted the error rates at very similar levels. The maximum differences, though very small, were at the extremes of read depths. At lower read depths, the noise estimates by the Poisson model were higher than those by the negative-binomial model, whereas at higher read depths, the opposite was the case. This is consistent with the assumption that errors originating during the initial PCR steps multiply exponentially to high levels and are represented by the gamma distribution. These error events remain inconspicuous when read depths are low but become apparent at high read depths, where they can be distinguished from the random error represented by the Poisson distribution. The near complete overlap of the error rates predicted by the Poisson and negative-binomial models indicates that minimal error occurs during the initial amplification steps of library preparation in this protocol.

### Variant analysis of vaccine-related polioviruses.

Based on these error data, we took a conservative approach for rare variant calling in 36 OPV-positive stool specimens from vaccinated children and their close contacts. First, we excluded five specimens in which nonpolio enterovirus C (NPEV-C) strains were detected. Then we evaluated only those OPV strains where 500× coverage was obtained over 95% of the 5′ UTR and P1 region (see Fig. S1 in the supplemental material). Given that several samples contained multiple serotypes, this allowed evaluation of 40 poliovirus sequences comprising 10 Sabin 1, 14 Sabin 2, and 16 Sabin 3 vaccine-related polioviruses. No evidence of recombination between serotypes was identified. The maximum time between the most recent national immunization week (NIW) and sample collection was 24 days for Sabin 1, 37 days for Sabin 2, and 66 days for Sabin 3 strains.

From these 40 sequences, variants from the raw variants files underwent the following informatics algorithm. First, positions with fewer than 500 total reads or fewer than 3 variant reads were discarded. Next, variants with ≥10% frequency were identified and considered true variants. For variants found within the error-prone region of Sabin 2 strains (positions 810 to 900), a fixed threshold of 5% was set for calling true variants. Lastly, for the remaining variants, thresholds were dynamically set based on read depth according to the Poisson function by using the observed error rate (0.0033) in control samples at a *P* of <0.001 (17). This resulted in the error threshold shown in Fig. 1D. For the 9 samples in which a second round of PCR amplification was required to generate enough amplicon for library preparation, a higher overall per base error rate of 0.0052 was used to calculate the error threshold. This per base error rate was obtained by subjecting the low-passage-number culture isolates to two rounds of amplification prior to sequencing and calculating the error threshold as described above.

**FIG 2:**
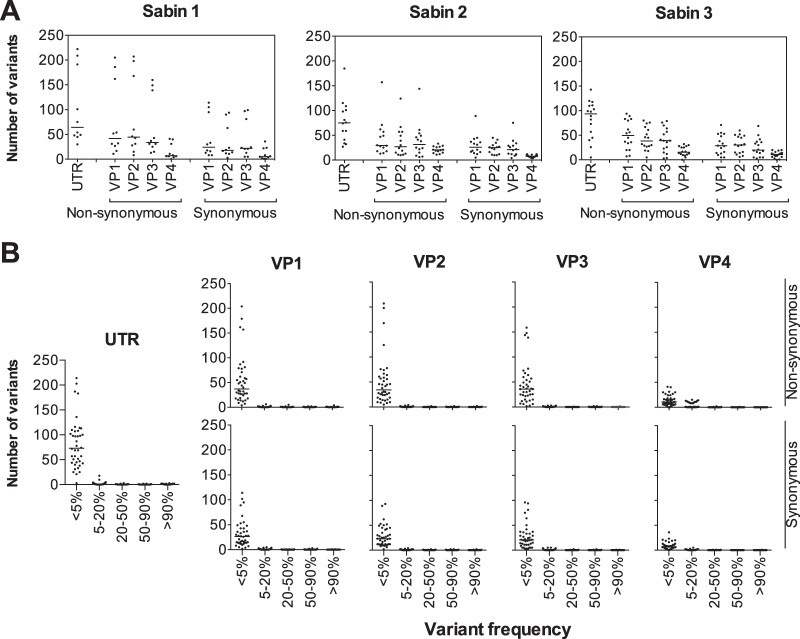
Variant analysis of vaccine-related polioviruses. (A) Distribution of synonymous and nonsynonymous variants in the 5′ UTR and VP region of Sabin 1, Sabin 2, and Sabin 3 vaccine-related polioviruses. Horizontal black bars, medians. (B) Distribution of variant abundance in the 5′ UTR and VP region. Horizontal black bars, medians.

The serotype distribution and frequency of sequence variants in the 5′-UTR and VP genes are shown in Fig. 2A and B. The median number of variants in Sabin1, Sabin 2, and Sabin 3 (medians, 255, 234, and 314; ranges, 98 to 1,112, 114 to 855, and 76 to 644; and interquartile ranges [IQRs], 875, 243, and 314, respectively) were not statistically different (Dunn's multiple-comparison test). Similarly, there was no statistical difference in the median number of variants in the 5′ UTR and VP1, VP2, and VP3 genes (medians, 79, 63, 61, and 59; ranges, 5 to 222, 12 to 319, 11 to 301, and 6 to 257; and IQRs, 64, 71, 68, and 68, respectively). The VP4 gene had significantly fewer variants than the 5′ UTR and other VP genes (median, 25; range, 0 to 67; IQR, 25; Kruskal-Wallis test, *P* < 0.001), though when normalized for sequence length, the number of variants was comparable across all VP genes and the 5′ UTR. Furthermore, normal mixture modeling accounting for the gene, read depth, and days from vaccination revealed that the virus strains showing a higher number of variants were outliers of a single population, rather than a separate population of high-rate mutators (data not shown).

The number of variants resulting in amino acid changes in the VP genes (medians, 41, 36, 38, and 17 for VP1 to VP4, respectively) was significantly higher (paired *t* tests, *P* < 0.001) than the number of synonymous variants (medians, 29, 25, 21, and 8 for VP1 to VP4, respectively). Of all variants, 95.6% (13,621/14,244) were detected at a frequency of less than the 5% level, and 66% (9,400/14,244) were detected at less than the 1% level. A total of 70 variants (0.5%) were above the 90% level, and 149 variants (0.86%) were above the 50% level.

**FIG 3:**
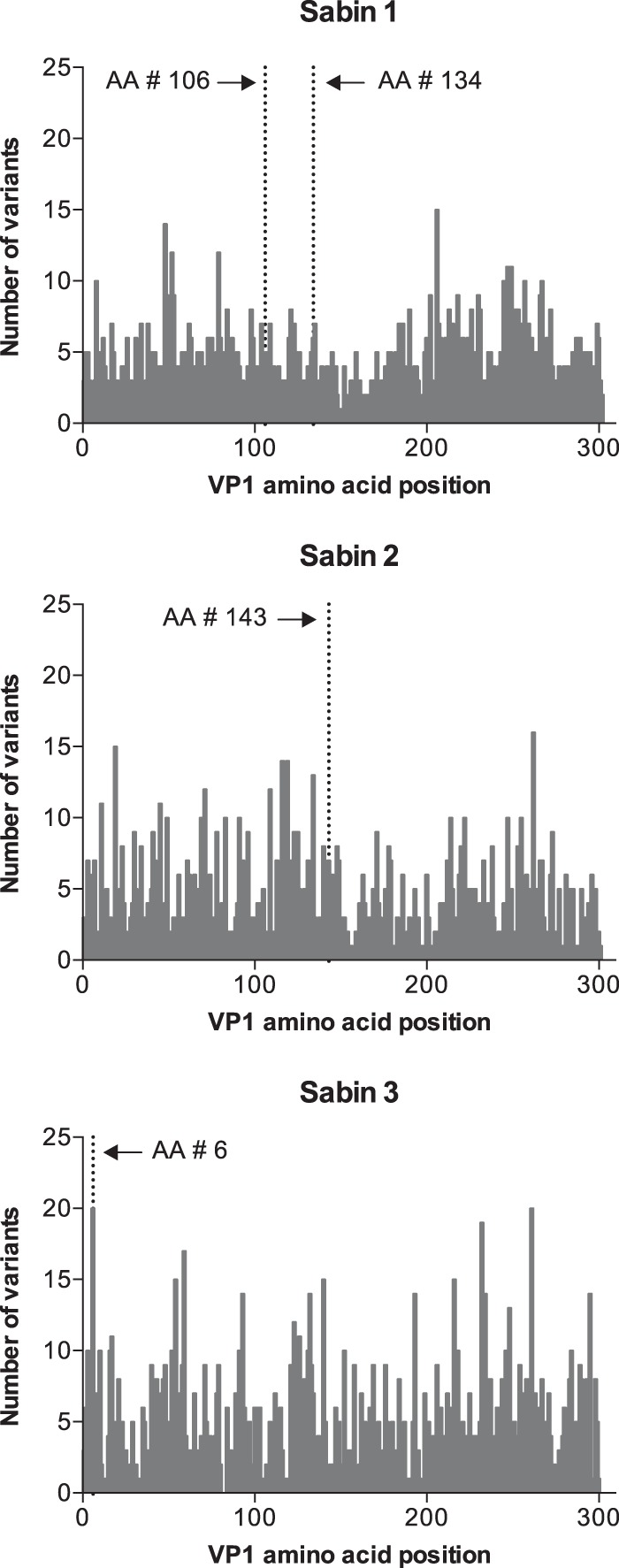
Frequency of VP1 nucleotide variants at each amino acid position in Sabin 1, Sabin 2, and Sabin 3. Dotted lines mark amino acid positions associated with phenotypic reversion. Variants include nonsynonymous and synonymous changes. AA, amino acid.

To evaluate for the emergence of VDPVs, VP1 sequences were further analyzed. Variants were distributed throughout the VP1 gene (Fig. 3). However, the median number of VP1 variants in Sabin 1 (median, 66; range, 19 to 319; IQR, 227), Sabin 2 (median, 56; range, 23 to 246; IQR, 68), and Sabin 3 (median, 75; range, 12 to 165; IQR, 99) were not statistically different. Though there were a large number of VP1 variants, 96% (3,430/3,556) were detected at a frequency of less than 5% and no VDPV strains were identified.

### Identification of canonical mutations associated with phenotypic reversion.

We next evaluated the ability of deep sequencing to identify canonical mutations associated with phenotypic reversion (reviewed in references 18 and 19) (Table 1). At least one of these mutations was found in all Sabin strains.

**TABLE 1:** Canonical OPV mutations associated with phenotypic reversion identified by deep sequencing^*a*^

Vaccine strain	Mutation(s) (amino acid change)^*b*^		
	5′ UTR	VP1	VP3
Sabin 1	G480A, T525C	A2795G (Thr106Ala), T2879C (Phe134Leu)	A2438T (Met225Leu)
Sabin 2	A481G	T2909C (Ile143Thr)	
Sabin 3	T472C	C2493T (Thr6Ile)	T2034C (Phe91Ser)

a Substitutions are from the reference Sabin sequence to the change associated with phenotypic reversion.

b Nucleotide changes are numbered according to the position in the complete genome. Amino acid changes (in parentheses) are numbered according to the position in the protein after polyprotein cleavage.

The 5′-UTR G480A mutation was found in 60% (6/10) of Sabin 1 strains. In five of these strains, the mutation was present in less than 15% of reads, but in one it was present in 26% of reads. Similarly, the 5′-UTR T525C mutation was found in 70% (7/10) of the Sabin 1 strains. In three of these strains, the mutation was present in less than 10% of reads, but in four it was present at greater than 20% of reads, including two at high levels (68% and 99%). The 5′-UTR A481G mutation was found in 100% (14/14) of Sabin 2 strains, with levels ranging from 0.5 to 100%. The 5′-UTR T472C mutation was found in 100% (16/16) of the Sabin 3 strains. This mutation was present in the majority (83 to 100%) of reads for 62.5% (10/16) of the Sabin 3 strains.

We also identified numerous strains with VP1 and VP3 mutations associated with phenotypic reversion. The VP1 Thr106Ala mutation was found in one Sabin 1 strain in 12% of reads. Other Sabin 1 mutations, including the VP1 Phe134Leu (3 strains) and VP3 Met225Leu mutations (3 strains) were found in ≤2.5% of reads. All of these VP1 and VP3 mutations were found in samples in which 5′-UTR mutations were identified, though the read length and mutation levels do not allow us to determine whether these mutations were present on the same virus genome.

The VP1 Ile143Thr mutation was found in three Sabin 2 strains. In two of these samples, where the VP1 mutation accounted for 70% and 2% of reads, the 5′-UTR mutation was present in >99% of reads, indicating that a subset of viruses contained both mutations.

For Sabin 3, 100% of the strains (16/16) had the VP1 Thr6Ile mutation in 69% to 100% of reads. In eight samples, this VP1 mutation and the 5′-UTR T472C mutation were found in >90% of reads, indicating that both mutations were frequently present in the same genome. Furthermore, the VP3 Phe91Ser mutation was present in seven of these strains, primarily at a low level (accounting for ≤4% of reads in six samples and 26% of reads in one), revealing that at least a small proportion of Sabin 3 genomes contained all three mutations.

## DISCUSSION

We present here a deep-sequencing method to detect and characterize OPV shed by vaccinated children and their close contacts. This involved the development of an informatics pipeline and error model to accurately distinguish low-level variants from noise, resulting in a sensitive, quantitative approach that can identify variants present at levels of <1% of the virus population.

This method represents the first application of deep sequencing to the study of vaccine-related polioviruses in stool specimens without culture amplification. While similar sequencing methods have been developed (20) and utilized to ensure the quality of vaccine preparations (21), as well as to describe the fitness landscape of serially passaged wild-type poliovirus type 1 Mahony (22), the evaluation of cultured virus in these studies ensured high concentrations of intact viral RNA that is not present in primary stool (23).

Nevertheless, we obtained high quality 5′-UTR and P1 sequences from 31 of 36 stool specimens, allowing sequence analysis of vaccine-related polioviruses without the bias of selection in culture. This analysis revealed the presence of large numbers of low-level synonymous and nonsynonymous variants throughout the 5′ UTR and P1 genomic region. In addition, mutations associated with phenotypic reversion were found in 39/40 (98%) sequenced vaccine-related polioviruses that underwent variant analysis, with many present at high levels. While these reversion mutations are known to increase virus fitness and be shed by healthy vaccinees, this deep-sequencing method allowed simultaneous evaluation of all known attenuating mutations and revealed the presence of viruses containing up to three canonical mutations associated with the reacquisition of neurovirulence (18, 19).

We demonstrated that VDPVs were not circulating in this primarily inactivated poliovirus vaccine (IPV)-vaccinated population with moderate to high OPV coverage rates. Characterization of sequence changes in the VP1 gene demonstrated a median of 63 changes per strain, including both nonsynonymous and synonymous changes, though 96% were found at levels of less than 5%. Twenty-two strains contained one or more VP1 variants at levels of >50%; however, no strains met the definition of a VDPV.

Work defining the poliovirus molecular clock has demonstrated that approximately 1% of nucleotides undergo substitution per year (24). The accumulation and fixation of nucleotide substitutions in VP1 to the levels that define VDPVs therefore require prolonged replication in an immunocompromised individual or person-to-person transmission for longer than 1 year (4, 19). We identified variants in 40 sequenced strains, and of these strains, the longest duration between sample collection and the end of the most recent NIW was 66 days. Thus, it is not surprising that no VDPVs were identified. However, VP1 mutations commonly found in VDPV outbreak strains were detected (25). These mutations alter amino acids in VP1 surface determinants and are subject to strong selection during viral replication in the intestine (26, 27). The presence of these VDPV-associated mutations coupled with identification of canonical mutations associated with phenotypic reversion suggests that this deep-sequencing method may be an important tool for the surveillance and early detection of VDPVs.

In nearly all cVDPV outbreaks, not only does the virus accumulate VP1 mutations and other mutations associated with phenotypic reversion, it also undergoes recombination with cocirculating nonpolio enterovirus C (NPEV-C) strains (4, 18, 19). These recombinant viruses are typically composed of vaccine-derived P1 sequences with some or all of the genes encoding the nonstructural proteins derived from an NPEV-C strain (4, 19). Study of recombinant cVDPV outbreak strains as well as laboratory-generated chimeric Sabin/NPEV-C viruses suggests that recombination may promote the emergence of VDPVs (28, 29). Interestingly, we detected five samples with both vaccine-related poliovirus and NPEV-C. Though recombination may also occur in the 5′ UTR (30, 31), we did not detect recombinants in the genomic regions interrogated by our sequencing assay. Future whole-genome approaches that evaluate the 5′ UTR and P1 region, as well as the nonstructural genes, will be required to detect recombinants and study their contribution to the development of VDPVs.

While the number of stool samples from which we were able to obtain sequences was sufficient to demonstrate the potential of this deep-sequencing method for the characterization of vaccine-related polioviruses, a larger sample size will be required to evaluate correlations between sequence variants and the likelihood of transmission and the development of VDPVs. In particular, sequencing OPV from closely spaced serial samples from vaccinees and their close contacts is necessary to describe the evolution of vaccine-related polioviruses into VDPVs and the determinants of this process.

In conclusion, we have developed a culture-independent, deep-sequencing method and informatics approach for the quantitative characterization of vaccine-related poliovirus variants. This method was successfully applied to primary stool specimens from trivalent OPV-vaccinated children and close household contacts collected during a prospective cohort study carried out in Veracruz, Mexico. Surveillance for emerging variant viruses and recombinants by deep sequencing may be an important tool to ensure the successful conclusion of the poliovirus endgame.

## MATERIALS AND METHODS

### Ethics.

This study was approved by the Stanford University Institutional Review Board.

### Study samples.

Samples were previously collected and deidentified (16, 32). We followed 72 children and 144 household contacts from four municipalities in or near Orizaba, Veracruz, Mexico in a prospective cohort study conducted from August 2010 to August 2011. Per Mexican national guidelines enacted in 2007, children receive IPV at 2, 4, 6, and 18 months of age. In addition, children 5 years old or younger who have received at least 2 doses of IPV are eligible to receive trivalent OPV during biannual national immunization weeks (NIW). At each of 12 monthly visits, stool was collected from the enrolled child and the household contacts and stored at −70°C. Stool samples were tested by a serotype-specific real-time RT-PCR that differentiates Sabin 1, 2, and 3 and detects canonical point mutations in the 5′ UTR that are associated with phenotypic reversion to neurovirulence (33).

### Control material.

Sabin serotype 1, 2, and 3 vaccine strains were cultured and RNA was extracted as described previously (33). Briefly, the vaccine strains were obtained from the laboratory of Konstantin Chumakov at the U.S. Food and Drug Administration (FDA) and were prepared and titrated according to the World Health Organization (WHO) Poliovirus Laboratory Manual (34).

### RNA extraction and DNA generation.

RNA was extracted from frozen stool samples on a QIAcube instrument using the RNeasy minikit (both from Qiagen, Germantown, MD) according to the manufacturer's instructions. Five microliters of isolated RNA was reverse transcribed for 1 or 5 h by SuperScript III reverse transcriptase (Thermo Fisher Scientific, Inc., Waltham, MA) according to the manufacturer's instructions using primer Q8R modified with a 5′ sequence tag (5′-*TACGGTAGCAGAGACTTGGTCT*AAGAGGTCTCTRTTCCACAT-3′). (The italicized sequence represents the 5′ sequence tag.) The ∼3.5-kb region encoding the viral structural proteins VP1 to VP4 along with the 5′ UTR was amplified using the modified primer Q8R, and primer UFP was also modified with a 5′ sequence tag (5′-*GCGGCCGCTAATACGACTCACTATAGG*TTAAAACAGCTCTGGGGTTG) (21, 35, 36). Each 50-μl reaction mixture contained LongAmp Hot Start *Taq* 2× master mix (New England BioLabs, Ipswich, MA), 400 nM each primer, 400 ng/μl bovine serum albumin (BSA), and 2 to 4 μl of cDNA. The reactions were performed in an Applied Biosystems Veriti 96-well thermal cycler, using the following cycling parameters: 94°C for 2 min; 40 cycles of 94°C for 30 s, 70°C for 1 s (ramp rate, 20%), 55°C for 45 s (ramp rate, 20%), and 65°C for 3 min 20 s; final extension for 10 min at 65°C. The PCR products were electrophoresed on a 1% agarose gel and visualized by ethidium bromide staining. The ∼3.5-kb amplicon (Sabin 1, 3,504 bp; Sabin 2, 3,503 bp; Sabin 3, 3,495 bp) was excised and purified using the QIAquick gel extraction kit (Qiagen, Germantown, MD). If the expected band was present but was too faint to yield sufficient DNA, the amplicon was utilized as the template for a second round of PCR. The purified PCR products were quantitated using the Qubit double-stranded DNA (dsDNA) high-sensitivity (HS) assay kit (Thermo Fisher Scientific Inc., Waltham, MA) in accordance with the manufacturer's instructions and stored in −20°C prior to library preparation.

The long-range RT-PCR product described above was cloned into pCR 2.1 Topo vector (Invitrogen, Grand Island, NY) according to the manufacturer's instructions. The templates used for cloning consisted of RNA from the Sabin 1 and Sabin 2 vaccine strains. Plasmids were purified using the GeneJET plasmid miniprep kit (Fermentas, Glen Burnie, MD) according to the manufacturer's instructions. The sequences of the cloned amplicons were confirmed using Sanger sequencing. DNA concentrations were measured using the Qubit dsDNA HS assay kit reagents. Plasmid dilutions were prepared at 100 copies/μl of eluate and were used as the template for long-range PCR and the subsequent assay steps in order to estimate error rates.

### Library preparation.

Fragment libraries were prepared from approximately 50 ng of PCR products using NEBNext DNA library prep reagents for Illumina (New England BioLabs, Ipswich, MA) per the manufacturer's instruction. DNA was fragmented using NEBNext dsDNA Fragmentase reagents, supplemented with 15 mM MgCl_2_ and incubated at 37°C for 30 min. The fragmentation reaction was stopped by adding 5 μl of 0.5 M EDTA (Ambion, Foster City, CA), and fragments were purified using Agencourt AMPure XP magnetic beads (Beckman Coulter, Brea, CA). Purified fragments were repaired and deoxyribosyladenine (dA) tailed using a NEBNext Ultra end repair/dA-tailing module. NEBNext adaptor for Illumina was ligated to the dA-tailed fragments using the NEBNext Ultra ligation module, and the adaptor was linearized by uracil-specific excision reagent enzyme in accordance with the manufacturer's instructions. Adaptor-ligated fragments were purified using AMPure XP magnetic beads, and the library was enriched by PCR using NEBNext high-fidelity PCR master mix and NEBNext multiplex oligonucleotides for Illumina. The following cycling conditions were used: 98°C for 10 s, 12 cycles of 98°C for 10 s, 65°C for 30 s, and 72°C for 30 s. This step also adds sample-specific barcodes.

The resulting PCR products were purified using AMPure XP magnetic beads and checked for quality on an Agilent 2100 expert bioanalyzer using the high-sensitivity DNA kit (Agilent Technologies, Santa Clara, CA). The bioanalyzer concentration estimate was used for the normalization of each sample library in the final pool of 24 samples for a single sequencing run. To eliminate any remaining adaptors or primer-dimers, the pooled library was purified using AMPure XP magnetic beads. Washing steps used a lower ethanol concentration (70%). The final library pool was checked for quality on the bioanalyzer as described above, and the library concentration was estimated using the Qubit dsDNA HS assay kit. The library concentration was calculated using the conversion factor 1 ng/μl = 6 nM per Illumina guidelines for libraries with ∼250-bp-sized fragments. The libraries were adjusted to concentrations of 4 nM before being used downstream.

### Illumina sequencing.

The pooled library was denatured using 0.2 N sodium hydroxide (Sigma-Aldrich, St. Louis, MO), diluted to 3 pM with hybridization buffer (Illumina, Inc., San Diego, CA) and mixed 80:20 with denatured 3 pM PhiX. The library was loaded into the Illumina flow cell, and 150-cycle paired-end sequencing was performed using MiSeq reagent kit v2 reagents on a MiSeq sequencer with MiSeq Control software version 2.4.1.3 (all from Illumina, Inc., San Diego, CA). FASTQ files were generated using the MiSeq reporter software (version 2.4.60.80).

### Alignment.

FASTQ files were aligned to full-length Sabin 1, 2, and 3 reference sequences (GenBank: V01150.1, AY184220.1, and AY184221.1, respectively) using Burrows-Wheeler Aligner's Smith-Waterman Alignment (BWA-SW) with default parameters (37). The BAM alignment files were checked visually using Tablet for coverage, and the count of reads was aligned to each reference (38). Potential recombinants were identified by evaluating the distribution of aligned reads and their start and end points, as described previously (39).

Sabin 1, 2, and 3 consensus sequences were generated, and variants were called by pileup function in SAMtools (40). In order to identify all variants before any noise filter was applied, a custom Python script using Pysam was used for variant calling (available upon request). A pileup table containing nucleotide base counts and events of deletions and insertions at each position along the reference sequence was generated from the alignment (BAM) file after filtering out poor-quality bases as represented by N in Tablet (38). For each position, any nonreference base with a count above zero was called and listed in the raw variants file.

### Error modeling and statistical analysis.

All variants found in low-passage-number culture isolates or plasmid clones were considered noise. At nucleotide positions where no variant was detected, the noise value was considered zero (41). Error rates for low-passage-number culture isolates and plasmid clones were calculated as the mean percent noise reported for all sequenced positions (42). The noise data were analyzed in R using the glm function to fit observed data into generalized linear models (43). Both Poisson and negative-binomial models were used to estimate the dependency of the number of noise reads on the following covariates: read depth, homopolymer or nonhomopolymer region, read orientation, and Sabin strain. Only read depth was kept in the final model because the number of noise reads did not depend strongly on all other variables (41, 44). A set of low-passage-number culture isolates were subjected to a second round of long-range PCR to represent the subset of samples which were amplified twice before library preparation when faint bands were observed in the first round. They were analyzed separately in the same manner.

Column statistics, the Kruskal-Wallis test, and Dunn's multiple-comparison test were performed using GraphPad Prism (GraphPad Software, Inc., La Jolla, CA). Normal mixture modeling was performed using the mclust package (version 5.2.2) in R (45).

## Supplementary Material

Supplemental material
