# Supplementary material for: Detection of Emerging Vaccine-Related Polioviruses by Deep Sequencing
Source: J Clin Microbiol. 2017 Jun 23;55(7):2162–71. doi: 10.1128/JCM.00144-17 (PMC5483918; doi:10.1128/JCM.00144-17)
Supplement: Supplemental material [file supp_55_7_2162__index.html]

Supplemental material 

# Detection of Emerging Vaccine-Related Polioviruses by Deep Sequencing

## Supplemental material

- Supplemental file 1 -

  Fig. S1 legend

  PDF, 45K
- Supplemental file 2 -

  Fig. S1 (Sequence coverage map)

  PDF, 3.8M
