# Supplementary material for: Detection of Emerging Vaccine-Related Polioviruses by Deep Sequencing
Source: J Clin Microbiol. 2017 Jun 23;55(7):2162–71. doi: 10.1128/JCM.00144-17 (PMC5483918; doi:10.1128/JCM.00144-17)
Supplement: Supplemental material [file JCM.00144-17_zjm999095566s1.pdf]

SUPPLEMENTAL MATERIAL

Supplemental Figure Legend

Figure S1. Sequence coverage map. Each curve represents the number of reads across each genomic position for one poliovirus strain. Sabin 1, 2, and 3 serotypes are displayed separately. Variants were called only when there were at least 500 reads (dotted line) across >95% of target positions in a given strain.
