# Supplementary figures and images for: Detection of Emerging Vaccine-Related Polioviruses by Deep Sequencing
Source: J Clin Microbiol. 2017 Jun 23;55(7):2162–71. doi: 10.1128/JCM.00144-17 (PMC5483918; doi:10.1128/JCM.00144-17)

Figure S1

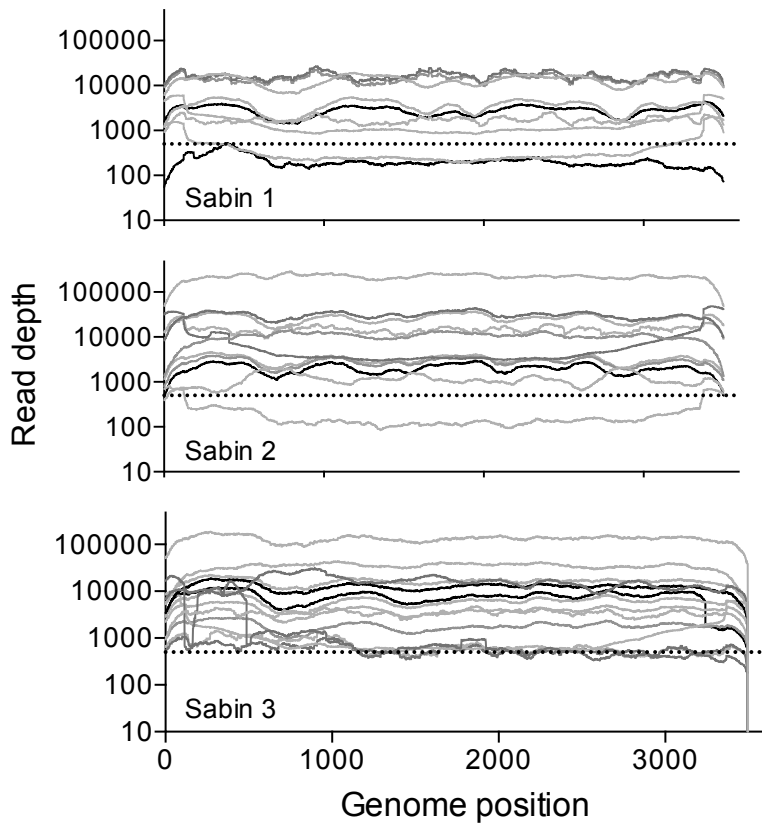

Supplement: Supplemental material [file JCM.00144-17_zjm999095566s2.pdf]
